# A Wi-Fi Indoor Positioning Method Based on an Integration of EMDT and WKNN

**DOI:** 10.3390/s22145411

**Published:** 2022-07-20

**Authors:** Rong Zhou, Fengying Meng, Jing Zhou, Jing Teng

**Affiliations:** School of Control and Computer Engineering, North China Electric Power University, Beijing 102206, China; zhourong@ncepu.edu.cn (R.Z.); 120202227098@ncepu.edu.cn (F.M.); jing.teng@ncepu.edu.cn (J.T.)

**Keywords:** RSSI fluctuation, EMD, WKNN, indoor positioning

## Abstract

In indoor positioning, signal fluctuation is one of the main factors affecting positioning accuracy. To solve this problem, a new method based on an integration of the empirical mode decomposition threshold smoothing method (EMDT) and improved weighted K nearest neighbor (WKNN), named EMDT-WKNN, is proposed in this paper. First, the nonlinear and non-stationary received signal strength indication (RSSI) sequences are constructed. Secondly, intrinsic mode functions (IMF) selection criteria based on energy analysis method and fluctuation coefficients is proposed. Thirdly, the EMDT method is employed to smooth the RSSI fluctuation. Finally, to further avoid the influence of RSSI fluctuation on the positioning accuracy, the deviated matching points are removed, and more precise combined weights are constructed by combining the geometric distance of the matching points and the Euclidean distance of fingerprints in the positioning method-WKNN. The experimental results show that, on an underground parking dataset, the positioning accuracy based on EMDT-WKNN can reach 1.73 m in the 75th percentile positioning error, which is 27.6% better than 2.39 m of the original RSSI positioning method.

## 1. Introduction

Due to non-line-of-sight obstacles such as roofs and walls, the global navigation satellite system (GNSS) fails to achieve desirable positioning in indoor environments [1]. With the emergence of a large number of indoor applications, scholars have conducted numerous studies. Indoor positioning technologies can be divided into two categories according to whether it requires dedicated infrastructure. Indoor positioning technologies that require dedicated infrastructures are radio frequency identification (RFID) [2], Bluetooth low energy (BLE) [3], light (invisible and infrared light [4]), sund (audible sound and ultrasonic [5]), ultra-wide band (UWB) [6] and others. Indoor positioning technologies that do not require dedicated infrastructures include wi-fi [7], computer vision [8], motion sensors [9], and so on. The type of positioning technology determines the method to obtain location. The common methods include the path loss distance model, angle of arrival (AOA), time of arrival (TOA), and fingerprint [10]. In infrared, the user transmits an infrared signal to an infrared receiver, and the TOA of the ultrasonic pulse can estimate the location from the transmitter to the receiver. In wi-fi, based on the received signal strength indication (RSSI) from wi-fi access points (AP), the location can be easily estimated by using the path loss distance model or the fingerprint methods. In addition, motion sensors can provide information about direction, speed, and acceleration. The location can be continuously updated by integrating the motion sensor information. Computer vision captures images from the user’s perspective and compares them with database images to estimate the user’s location.

Among these wireless systems, wi-fi fingerprint positioning is favored in indoor positioning because most mobile devices have the function of receiving wi-fi signals, and APs are widely deployed indoors. The wi-fi fingerprint positioning method has its advantages: no additional hardware, easy deployment, wide coverage, and low cost [11]. In wi-fi fingerprinting, the reference point (RP) or test point (TP) receives the RSSIs from each AP, and these RSSIs compose the fingerprint. The main idea of wi-fi fingerprint positioning is to match the TP fingerprint with the RP fingerprint in the fingerprint dataset and predict the coordinates of the TP according to the matched RP coordinates.

However, the RSS fluctuation is one of the important reasons that lead to the severe degradation of indoor positioning system performance. In the actual environment, due to the complex and changeable indoor environment, other equipment interferences, and multipath effects, the RSSI values fluctuate [12]. Furthermore, due to changing environment, it is difficult for APs to transmit signals with a fixed power, which leads to the time-varying RSSI [13]. Therefore, the RSSI sequence has complex nonlinear and non-stationary characteristics.

The mean filter and Gaussian filter are classic methods to address RSSI fluctuation. However, the mean filter does not have high confidence when dealing with sharp fluctuation in RSSI. The Gaussian filter can also reduce the impact of noise with small probability and strong interference. Still, RSSI does not strictly conform to the normal distribution, and there are multi-peaked distribution states or skewed distribution [14]. The authors in [15] set the upper limit of signal fluctuation T according to the RSSI value. When the RSSI difference between TP and RP is greater than T, the RSSI difference between TP and RP is recorded; otherwise, the RSSI difference is set to 0. But the value of T is difficult to choose. Considering the insufficiency of a single filter, the authors in [16] proposed a moving mean-Kalman filter. The filter sets a mean value and its borders, and if a new RSSI is over the specified range, the new RSSI is assigned to the mean RSSI value. The authors in [17] proposed the particle filter-extended Kalman filter, which first uses the particle filter to obtain the user location, then uses the extended Kalman filter to smooth the user location, thereby reducing the fluctuation in location estimate resulting. Although these filtering methods improved the positioning accuracy, these methods only alleviate linear or nonlinear fluctuation and cannot effectively deal with the dynamic changes of the indoor environment. Another method to deal with RSSI fluctuation is based on the relationship between RSSIs. Based on the spatial correlation of RSSIs measured at adjacent RPs and the temporal correlations of RSSIs measured at the same RP at different times, a low-rank fingerprint dataset was constructed to remove the outliers and noise [18]. Because the dynamic environment and device differences have almost the same impact on the RSSI value, a robust NS-RSS fingerprint based on the RSSI differences between adjacent RPs was constructed to eliminate RSSI fluctuation due to the environment and device differences [19]. These reconstructed fingerprint datasets based on the relationship RSSIs require complex calculations. In addition, the authors in [20] measured the noise floor in different environments to mitigate RSSI fluctuation caused by noise. But this method is not quite feasible in a real environment, not only as the noise floor has to reset every time, but also because the noise floor varies greatly in different time periods in the same environment. Alternatively, some solutions to RSSI fluctuation are developed based on machine learning. The authors in [21] used a singular value decomposition (SVD) method to suppress noise-related subspaces to smooth RSS fluctuation. The authors in [22] proposed a convolutional neural network (CNN) model to extract the RSSI fluctuation patterns and learn the nonlinear mappings from the RSSI features. However, to achieve good performance, RSSI smoothing methods based on machine learning require a large amount of RSSI data.

According to the signal propagation model, our previous work [23] proposed a Q-based RSS transformation to smooth RSSI fluctuation. This method is more advantageous when the RSSI fluctuates sharply due to environmental changes. But Q value is an empirical parameter and cannot be dynamically adjusted according to the real environment. Therefore, we propose an empirical mode decomposition [24] threshold smoothing method (EMDT) to smooth RSSI fluctuation. The EMDT does not need pre-determined basis functions as it derives basis functions from the data itself [25].

In this paper, the EMDT is adopted to deal with RSSI fluctuation while using the improved weighted K nearest neighbor (WKNN) method is used to revise positioning accuracy further. The main contributions of this paper are listed as follows.

- To deal with RSSI fluctuation, the RSSIs need to be integrated into nonlinear and non-stationary RSSI sequences. Then an EMD method for adaptively decomposing the RSSI sequence is proposed.
- We set the fluctuation coefficients of intrinsic mode functions (IMF) that can reflect the degree of IMF fluctuation. Then new criteria of IMF selection are proposed based on energy analysis and fluctuation coefficients. The method divides IMFs decomposed by EMD into the fluctuation-domain IMFs (FD-IMF) and the effective IMFs (E-IMF) according to the characteristics of IMFs.
- An improved WKNN method is proposed: a secondary selection method is used to remove the matching RPs far from the geometric center of the K initial matching RPs. The Euclidean distance of the matching RPs and the Euclidean distance of fingerprints are combined to obtain more precise weights. The improved WKNN avoids the deviated matching RPs due to RSSI fluctuation and further corrects the positioning accuracy by combined weights.

The positioning experiment was carried out on the underground parking dataset of North China Electric Power University. The experimental results show that the indoor positioning algorithm based on EMDT-WKNN increased the positioning accuracy.

The subsequent sections of this paper are organized as follows. Section 2 provides a brief description of EMD and indoor positioning principle. Section 3 details the construction rules for RSSI sequences and the proposed EMDT-WKNN. In Section 4, the roles of EMDT and WKNN on RSSI fluctuation are demonstrated, and indoor positioning experiments are conducted to verify the improvement of positioning accuracy by EMDT-WKNN. Finally, Section 5 concludes with a summary of the conclusions.

## 2. Related Work

### 2.1. EMD

The EMD, first introduced by Huang et al., is a time-frequency signal decomposition tool that is useful to analyze nonlinear and non-stationary data [25]. The EMD algorithm assumes that any signal consists of different intrinsic modes of oscillations, and the EMD can adaptively decompose any complex signal into a set of IMFs from high to low frequencies and a residual function. For each IMF, it must satisfy the following stopping criteria: first, the number of local extrema and the number of zero-crossings differ at most by one; second, the upper envelopes defined by local maxima and the lower envelopes defined by local minima are locally symmetric with the time axis [26].

The procedure of EMD adaptive decomposition of a signal X(t) is as follows.

1. Find out all the local maxima in X(t), and interpolate them to form an upper envelope. In the same way, form a lower envelope according to all the local minima.
2. Calculate the mean envelopes m(t) by averaging the upper and lower envelopes.
3. Calculate a temporary local oscillation h(t):
  (1) h(t)=X(t)−m(t).
4. If h(t) meets the IMF stopping criteria, then obtain the first IMF: $imf_{1}\left(t\right)=h\left(t\right)$, otherwise repeat Steps (1) to (2) for h(t) until $imf_{1}\left(t\right)$ is obtained.
5. Calculate the residue $r_{1}\left(t\right)$:
  (2) $$r_{1}\left(t\right)=X\left(t\right)-imf_{1}\left(t\right).$$
6. Repeat Steps (1) to (5) by using $r_{1}\left(t\right)$ to obtain $imf_{2}\left(t\right),imf_{3}\left(t\right),\ldots ,imf_{n}\left(t\right)$ until $r_{n}\left(t\right)$ approaches zero or shows a monotonic trend.

**Figure 1:**
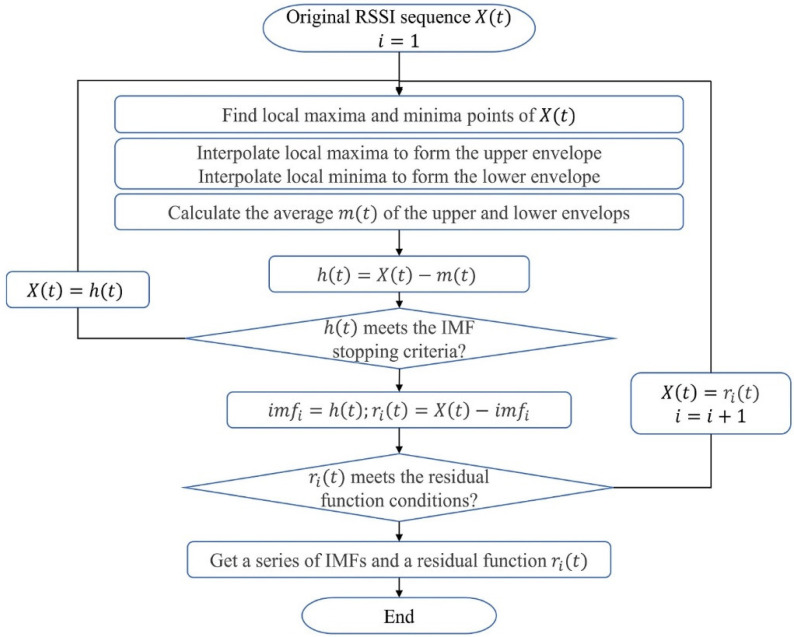
The flow chart of the EMD algorithm.

After EMD-based decomposition, the original signal X(t) can be represented as follows:

(3) $$X\left(t\right)=\sum_{i=1}^{n}imf_{i}\left(t\right)+r_{n}\left(t\right),$$

where i=1,2,…n is the number of IMFs; $r_{n}\left(t\right)$ is the residual function. The flow chart of the EMD algorithm is shown in Figure 1.

### 2.2. Fingerprint Positioning Principle

**Figure 2:**
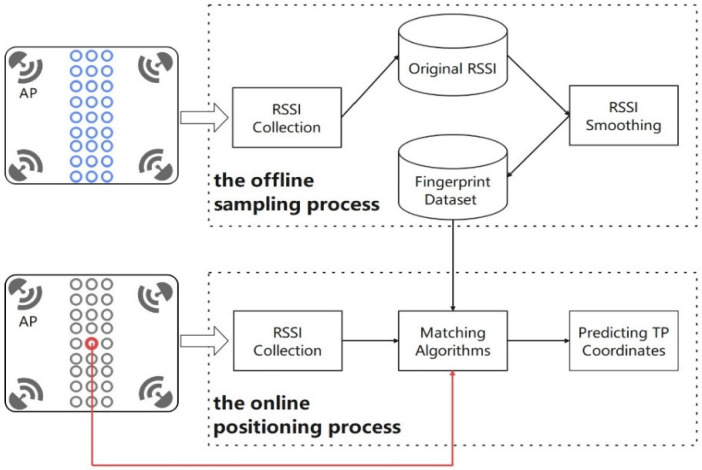
The flow chart of WIFI fingerprint positioning.

The wi-fi fingerprint positioning is mainly divided into offline sampling and online positioning processes. In the offline sampling process, the main task is to construct a location fingerprint database. The RP fingerprint is composed of the RSSIs from different APs measured at the same RP; the fingerprint database is composed of RP fingerprints and RP coordinates. In the online positioning process, the main work is to predict the TP coordinates: match the fingerprint measured at TP with the fingerprint database according to a matching algorithm, and predict the TP coordinates based on the matching RPs [27]. The flow chart of wi-fi fingerprint positioning is shown in Figure 2.

Suppose $AP_{i}$ represents the i-th AP, $RP_{j}$ represents the j-th RP, and $RSSI_{AP_{i}}^{RP_{j}}$ represents the RSSI from $AP_{i}\;$ measured at $\;RP_{j}$. The numbers of APs and RPs are n and m, respectively. The $TP_{j}$ fingerprint is shown in Equation (4) and the $RP_{j}$ fingerprint is shown in Equation (5):

(4) $$FP_{TP_{j}}=\left(\begin{array}{ccc} RSSI_{AP_{1}}^{TP_{j}} & RSSI_{AP_{2}}^{TP_{j}} & \ldots \;\;RSSI_{AP_{n}}^{TP_{j}} \end{array}\right)$$

(5) $$FP_{RP_{j}}=\left(\begin{array}{ccc} RSSI_{AP_{1}}^{RP_{j}} & RSSI_{AP_{2}}^{RP_{j}} & \ldots \;\;RSSI_{AP_{n}}^{RP_{j}} \end{array}\right)$$

$RP_{j}$ has coordinates $\left(x_{j},y_{j}\right)$ and the fingerprint database includes the fingerprint and coordinates of $RP_{j}$:

(6) $$FPDB_{i}=\left(\begin{array}{ccc} RSSI_{AP_{1}}^{RP_{j}} & RSSI_{AP_{2}}^{RP_{j}} & \ldots \end{array}\;\;\;\;\;\begin{array}{ccc} RSSI_{AP_{n}}^{RP_{j}} & x_{j} & y_{j} \end{array}\right).$$

## 3. The Proposed Method

### 3.1. RSSI Sequence

**Figure 3:**
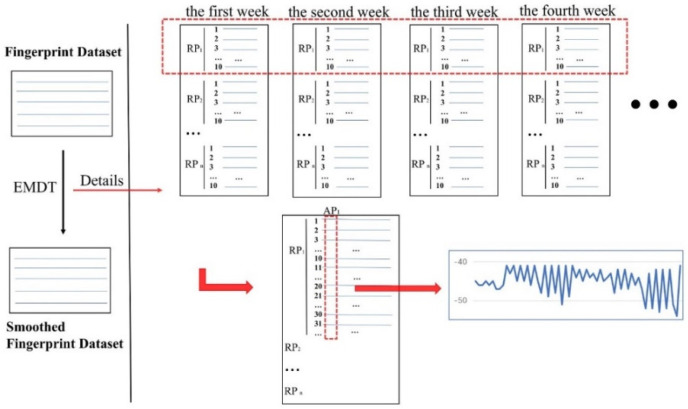
The flow chart of integrating the training RSSI.

Before using EMD to decompose the RSSI sequence, it is necessary to construct a time-based nonlinear and non-stationary RSSI sequence. In this paper, the nonlinear and non-stationary RSSI sequence is constructed according to the RSSIs from a single AP measured at a single RP. Because the amount of fingerprint data measured by each time on each RP is too small in actual measurement, it is necessary to integrate the fingerprint data measured each time. Therefore, to obtain the RSSI sequences, all the fingerprint data are integrated according to the RP, and then the fingerprint data is integrated according to the AP. Figure 3 is the flow chart of integrating RSSIs from $AP_{1}$ measured at $RP_{1}$.

In Figure 3, the upper red rectangle indicates the integration of all weeks of fingerprints measured at $RP_{1}$; the lower red rectangle indicates that according to the integrated $RP_{1}$ fingerprint data, the RSSIs from $AP_{1}$ are selected to construct nonlinear and non-stationary RSSI sequence. The curve graph visualizes the RSSI sequence, with the horizontal axis being the number of measuring times and the vertical axis being the RSSI value.

### 3.2. EMDT

EMDT is a data reconstruction method. The EMDT consists of four main steps: decomposing RSSI sequence by EMD, dividing IMFs into FD-IMFs and E-IMFs by IMF selection criteria, performing soft threshold processing on the FD-IMFs, and reconstructing the RSSI sequence by the processed FD-IMFs, E-IMFs, and residual function. In the following section, the IMF selection criteria based on energy analysis method and fluctuation coefficients, and the construction of the smoothed RSSI will be described in detail.

#### 3.2.1. IMF Selection Criteria

After EMD decomposition, the RSSI sequence X(t)  becomes multiple IMFs and a residual function. The frequency of each IMF decreases as the order number of the IMF increases, and the fluctuating components of X(t) are mainly distributed in the high-frequency IMFs [28]. Thus, these IMFs can be divided into two groups: FD-IMFs and E-IMFs. The FD-IMFs are high-frequency IMFs that are usually used to represent noisy data and fluctuating information; the E-IMFs mostly are low-frequency IMFs. The E-IMFs and the residual functions are usually used to represent features of the original signal. The result above can be expressed as:

(7) $$X\left(t\right)=\overset{j}{\underset{i=1}{\sum }}imf_{i}\left(t\right)+\overset{n}{\underset{i=j+1}{\sum }}imf_{i}\left(t\right)+r_{n}\left(t\right)$$

where the $imf_{1},imf_{2},\ldots ,imf_{j}$ are FD-IMFs; the $imf_{j+1},imf_{j+2},\ldots ,imf_{n}$ are E-IMFs; j is the boundary of FD-IMFs and E-IMFs.

It is very important to determine the boundary j. The traditional energy analysis [29] method uses the energy transfer model to estimate the possible fluctuation-only energy in $imf_{i}$. If the possible fluctuation-only energy in $imf_{i}$ is below the fluctuation energy of $imf_{i}$. The $imf_{i}$ is regarded as E-IMF. However, the energy transfer model is estimated by analyzing the characteristics of the EMD decomposed Gaussian white noise, and the characteristics of fluctuation in the actual signal are usually unknown [30]. In addition, the parameters of the energy transfer model require manual intervention. This paper sets a coefficient, which is determined according to the characteristics of the IMF itself, and the coefficient can reflect the fluctuation of IMFs. In this paper, the coefficient is named as fluctuation coefficient.

On the one hand, the IMF energy analysis method is to compare the fluctuation energy of IMFs with the possible fluctuation-only energy. The $imf_{1}$ is a high-frequency component that contains the most fluctuation, and the fluctuation energy of $imf_{1}$ can be used as a benchmark [31]. On the other hand, the fluctuation coefficient is constructed by using the standard deviation and the fluctuation standard deviation for each IMF. The fluctuation coefficient can reflect the fluctuation degree of IMFs, and the larger the coefficient, the smaller the fluctuation in the IMF. The possible fluctuation-only energy of each IMF is estimated by using the fluctuation coefficient and the fluctuation energy of $imf_{1}$. Find the first $imf_{i}$ that the fluctuation energy is less than the possible fluctuation-only energy, and take this $imf_{i}$ as the boundary, consider $imf_{2},imf_{3},\ldots ,imf_{i-1}$ as FD-IMFs, and $imf_{i},imf_{i+1},\ldots ,imf_{n}$ as ED-IMFs. The details of the improved energy analysis method are described as follows.

1. Estimate the standard deviation ${\hat{\sigma }}_{i}$ of the fluctuation in $imf_{i}$ by using a robust estimator [32] based on the IMF median
  (8) $${\hat{\sigma }}_{i}=\frac{median\left(\left|imf_{i}\left(t\right)-\bar{imf_{i}\left(t\right)}\right|\right)}{0.6745},\;i=1,2,\ldots n;t=1,2,\ldots ,N$$
  where i=1,2,…,n  is the number of IMFs; t=1,2,…N is the sampling point of X(t).
  (9) $$\bar{imf_{i}\left(t\right)}=\frac{1}{N}\overset{N}{\underset{t=1}{\sum }}imf_{i}\left(t\right)$$
2. Calculate the fluctuation energy $E_{i}$ of the $imf_{i}$:
  (10) $$E_{i}={\hat{\sigma }}_{i}{}^{2},i=1,2,\ldots ,n.$$
3. Calculate the standard deviation $\sigma_{i}$ of $imf_{i}$:
  (11) $$\sigma_{i}=\sqrt{\frac{1}{N-1}\sum_{n=1}^{N}{\left(imf_{i}\left(t\right)-\bar{imf_{i}\left(t\right)}\right)}^{2}}\;,i=1,2,\ldots n;t=1,2,\ldots ,N.$$
4. Construct the fluctuation coefficient $K_{i}$ of the $imf_{i}$:
  (12) $$K_{i}=\frac{\frac{\sigma_{i}}{{\hat{\sigma }}_{1}}+\frac{{\hat{\sigma }}_{i}}{\sigma_{i}}}{2},i=1,2,\ldots ,n.$$
5. Estimate the possible fluctuation-only energy according to the fluctuation coefficient and the fluctuation energy of $imf_{1}$. The possible fluctuation-only energy ${\hat{E}}_{i}$ of the $imf_{i}$ is approximately as
  (13) $${\hat{E}}_{i}=K_{i}\times E_{1},i=1,2,\ldots ,n.$$

The FD-IMFs are chosen by comparing the fluctuation energy $E_{i}$ of each IMF with the possible fluctuation-only energies ${\hat{E}}_{i}$. If $E_{i}<{\hat{E}}_{i}$ and $E_{i-1}>{\hat{E}}_{i-1}$, the $imf_{2},imf_{3},\ldots ,imf_{i-1}$ are judged to be FD-IMFs, and $imf_{i},imf_{i+1},\ldots ,imf_{n}$ are judged to be E-IMFs.

#### 3.2.2. Threshold Smoothing

After dividing IMFs into FD-IMFs and E-IMFs, threshold-based smoothing techniques are used to remove fluctuation inherent in FD-IMFs. For threshold-based smoothing techniques, two types of thresholding operators have been used for the processing of FD-IMFs: hard thresholding and soft thresholding [33]. The mathematical expression of the hard threshold method is defined as

(14) $$\overset{˘}{imf_{i}}\left(t\right)=\{\begin{array}{c} imf_{i}\left(t\right),\left|imf_{i}\left(t\right)\right|\geq TH_{i}\\ 0,\left|imf_{i}\left(t\right)\right|<TH_{i} \end{array}$$

(15) $$TH_{i}=C\sqrt{2E_{i}lnN},$$

where $\overset{˘}{imf_{i}}\left(t\right)$ is the smoothed version of $imf_{i}\left(t\right)$, $TH_{i}$ is the threshold of $imf_{i}\left(t\right)$, N is the number of data samples of the RSSI sequence X(t), C is an empirical constant that makes the $TH_{i}$ more flexible. In this study, C is set to 0.5 [34]. The mathematical expression of the soft threshold method is defined as

(16) $$\overset{˘}{imf_{i}}\left(t\right)=\{\begin{array}{c} sign\left(imf_{i}\left(t\right)\right)\times \left(\left|imf_{i}\left(t\right)\right|-TH_{i}\right),\left|imf_{i}\left(t\right)\right|\geq TH_{i}\\ 0,\left|imf_{i}\left(t\right)\right|<TH_{i} \end{array}$$

(17) $$sign\left(x\right)=\{\begin{array}{c} 1,x>0\\ -1,x<0 \end{array}.$$

Because the hard thresholding method may lead to the smoothed signal discontinuity [35]. Moreover, there may be useful information in FD-IMFs, and discarding FD-IMFs will cause a loss of useful information. For these deficiencies, this paper uses the soft threshold method to deal with FD-IMFs.

Finally, the FD-IMs after soft thresholding, ED-IMFs, and the residual function are reconstructed to obtain the smoothed RSSI sequence

(18) $$X_{f}\left(t\right)=\overset{j}{\underset{i=1}{\sum }}{\overset{˘}{imf}}_{i}\left(t\right)+\overset{n}{\underset{i=j+1}{\sum }}imf_{i}\left(t\right)+r_{n}\left(t\right)\;$$

where $X_{f}\left(t\right)$ is the smoothed RSSI sequence.

Based on the characteristics of the signal itself, the $X_{f}\left(t\right)$ can filter out fluctuation data of X(t), retain the local characteristics of X(t), and effectively smooth fluctuation.

**Figure 4:**
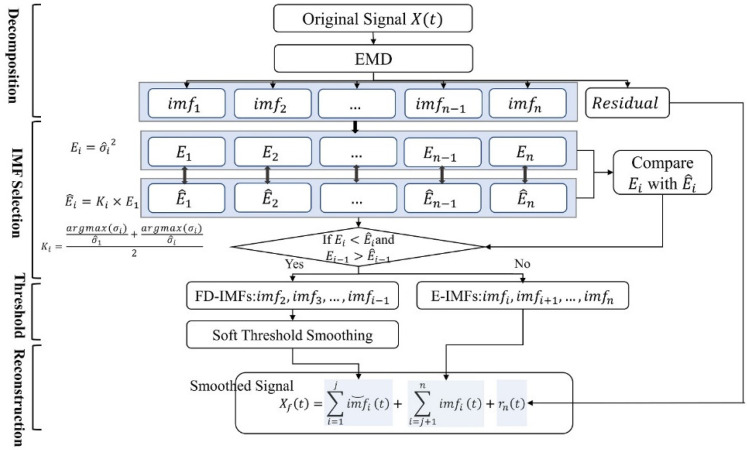
Schematic diagram of EMDT.

According to the above description, the EMDT contains four steps: RSSI sequence decomposition by EMD, IMF selection, FD-IMFs soft threshold, and smoothed RSSI reconstruction. Figure 4 shows the schematic diagram of EMDT.

### 3.3. Improved WKNN

The common fingerprint positioning algorithm is based on the KNN algorithm. The KNN positioning algorithm finds the K RPs the most similar to the TP fingerprint in the fingerprint dataset and uses the average coordinates of the K RPs as the TP prediction coordinates. As an improvement of the KNN algorithm, the WKNN positioning algorithm calculates the weights of each RP according to the similarity between the RP fingerprint and the TP fingerprint, and then predicts TP coordinates according to the Equation (19):

(19) $$\left(\hat{x},\hat{y}\right)=\overset{K}{\underset{i=1}{\sum }}W_{i}*\left(x_{i},y_{i}\right),\left(K\geq 2\right)$$

(20) $$W_{i}=\frac{\frac{1}{d_{i}}}{\sum_{i=1}^{K}\frac{1}{d_{i}}}$$

(21) $$d_{i}=\sqrt{\sum_{l=1}^{n}{\left(RSSI_{AP_{l}}^{RP_{i}}-RSSI_{AP_{l}}^{TP_{j}}\right)}^{2}},i=1,2,\ldots ,K,$$

where i=1,2,…,K is the number of RPs, $d_{i}$ is the Euclidean distance between $TP_{j}$ and $RP_{i}$ fingerprints, and is named fingerprint similarity metric, $W_{i}\;$ is the weight of $RP_{i}$; $\left(x_{i},y_{i}\right)$ is the $RP_{i}$ coordinates, and $\left(\hat{x},\hat{y}\right)$ is the $TP_{j}$ predicted coordinates.

However, due to the RSSI fluctuation, there may be some deviated matching RPs in the K initial matching RPs. By using these RPs to predict the $TP_{j}$ coordinates will directly affect the positioning result. The improved WKNN fingerprint positioning algorithm proposed in this paper:

1. Obtain the K initial matching RPs by WKNN: $RP_{1},RP_{2},\ldots ,RP_{K}$.
2. Geometry analysis of the initial matching RPs, calculating the Euclidean distance $d_{\;}{}_{ic}$ between $RP_{i}$ coordinates and the center coordinates $\left(x_{c},y_{c}\right)$.
  (22) $$\left(x_{c},y_{c}\right)=\frac{1}{K}\overset{K}{\underset{i=1}{\sum }}\left(x_{i},y_{i}\right)$$
  (23) $$d_{ic}=\sqrt{{\left(x_{i}-x_{c}\right)}^{2}+{\left(y_{i}-y_{c}\right)}^{2}},i=1,2,\ldots K.$$
3. Secondary selection: setting a threshold D, and if $d_{ic}>D$, the $RP_{i}$ is judged to be a deviated point and should be removed, finally obtaining the $K^{\prime }$ RPs with the closest distance from the $\left(x_{c},y_{c}\right)$. The value of D is discussed in Section 4.
4. Calculate the center coordinates $\left(x_{c}^{\prime },y_{c}^{\prime }\right)$ and Euclidean distance $d_{ic}^{\prime }$,
  (24) $$\left(x_{c}^{\prime },y_{c}^{\prime }\right)=\frac{1}{K^{\prime }}\overset{K^{\prime }}{\underset{i=1}{\sum }}\left(x_{i},y_{i}\right)$$
  (25) $$d_{ic}^{\prime }=\sqrt{{\left(x_{i}-x_{c}^{\prime }\right)}^{2}+{\left(y_{i}-x_{c}^{\prime }\right)}^{2}},i=1,2,\ldots ,K^{\prime }.$$
5. Combined weight: obtaining the combined weight $W_{i}^{\prime }$ according to fingerprints similarity metric $d_{i}$ and coordinates Euclidean distance $d_{ic}^{\prime }$,
  (26) $$W_{i}^{\prime }=\frac{\frac{1}{d_{ic}^{\prime }}+\frac{1}{d_{i}}}{\sum_{i=1}^{K^{\prime }}d_{ic}^{\prime }+\sum_{i=1}^{K^{\prime }}d_{i}}\;.$$
6. Predict $TP_{j}$ coordinates $\left(\hat{x},\hat{y}\right)$
  (27) $$\left(\hat{x},\hat{y}\right)=\sum_{i=1}^{K^{\prime }}W_{i}^{\prime }*\left(x_{i},y_{i}\right),\left(K^{\prime }\geq 2\right)\;.$$

**Figure 5:**
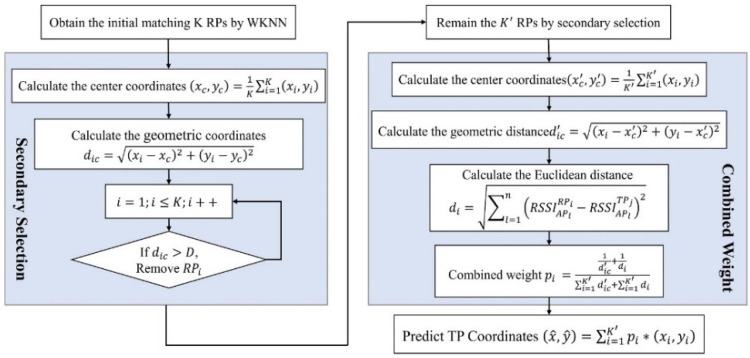
The flow chart of the improved WKNN.

Figure 5 is the flow chart of the improved WKNN fingerprint positioning algorithm. The improved WKNN algorithm is divided into two aspects. The first is to use the secondary selection method to remove the deviated matching RPs in the K initial matching RPs. The second is to construct combined weights from the fingerprint similarity metric and the Euclidean distance of matching RPs. The combined weights mitigate the impact of RSSI differences on positioning accuracy.

### 3.4. EMDT-WKNN

In order to mitigate the problem of RSSI fluctuation affecting indoor positioning accuracy, this paper deals with RSSI fluctuation in the offline sampling process and the online positioning process, respectively. In the offline sampling process, the nonlinear and non-stationary RSSI sequences are constructed, and the EMDT method is used to smooth the RSSI sequences to reduce or eliminate the influence of environmental factors on the RSSI. Then the smoothed RSSI is stored in the fingerprint database. The EMDT method can not only simply and effectively eliminate the fluctuation of RSSI but also retain the characteristics of RSSI.

**Figure 6:**
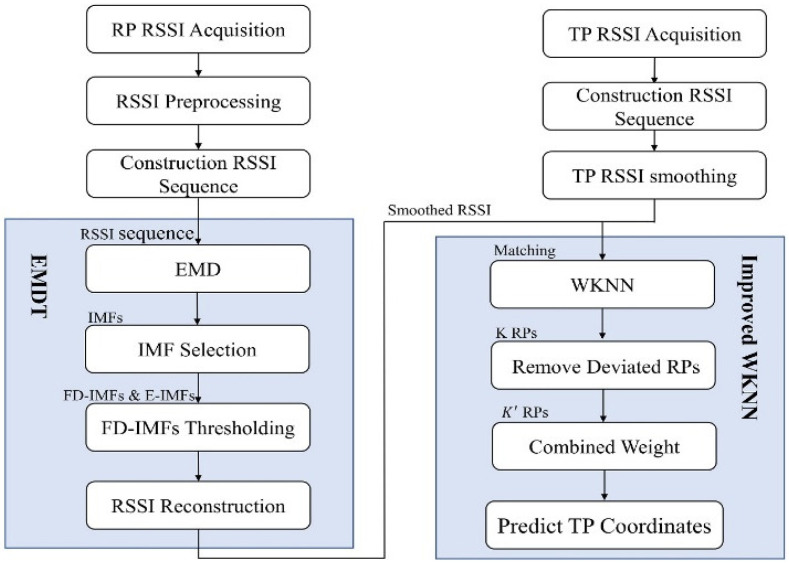
The framework of the EMDT-WKNN-based indoor positioning method.

In the online positioning process, the WKNN method is used to obtain the K initial matching RPs, a secondary selection is performed to remove the matching RPs that are far from the center of the K initial matching RPs, and then combined weights are obtained by combining the Euclidean distance of the matching RPs and the fingerprint similarity metric. Finally the TP location is predicted by the retained matching RPs after the secondary selection and combined weights. Figure 6 is an indoor positioning framework based on EMDT-WKNN.

## 4. Discussion

### 4.1. Experimental Environment

**Figure 7:**
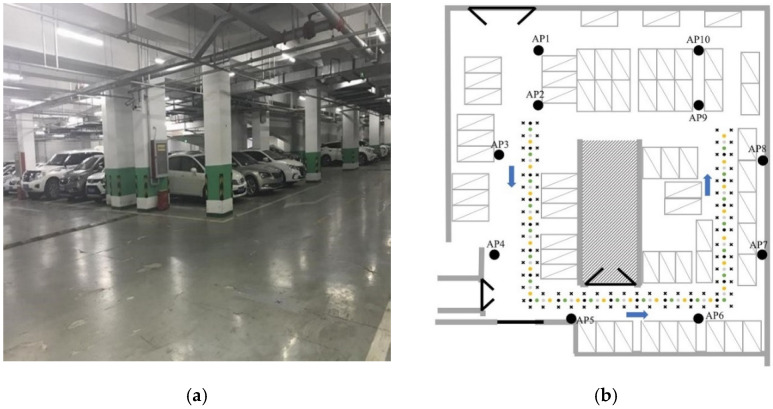
Experimental environment. (**a**) The actual scene of the underground parking lot. (**b**) Structural diagram of the experiment area.

In order to verify the feasibility of the EMDT-WKNN, this paper conducts a positioning experiment in the underground parking lot of North China Electric Power University. Figure 7a shows the actual scene of the underground parking lot. The area is about 58 m long, 42 m wide, and 5 m high. The experimental environment contains the entrance, walkways, and parking spaces of the underground parking lot. Figure 7b is a structural diagram of the experiment area, and the direction indicated by the blue arrow in the walkways area is the RSSI collection direction. As shown in Figure 7b, a total of 10 APs (the black ellipses) are evenly arranged in the experimental site, which are installed at the height of 2 m from the ground. The 10 APs are routers of different brands, and each AP transmits signals in two frequency bands, 2.4 GHz and 5 GHz. In walkways area, the “x” represents RP, and the “o” represents TP. These RPs are divided into two groups of training sets according to the outside (45 RPs) and inside (41 RPs), and the distance between adjacent RPs in each group is 2 m; TP is divided into four groups of test sets according to color (black, green, grey, yellow), and each test set has 21 TPs, and the distance between same color TPs is 4 m.

The experiment spanned 3 months, with 6 weeks of fingerprint data. The experimenters measured four weeks of data during the first month and one week of data each month thereafter. Because the number of vehicles in the underground parking lot in the afternoon is 2 to 3 times that in the evening, vehicles are not only obstacles that affect signal propagation, but also devices such as CarLog, in-vehicle wi-fi, and Bluetooth interfere with the AP’s WIFI signal. Therefore, RP and TP in each group were measured weekly in the afternoon and evening, respectively, and the weekly collection order is shown in Table 1. The weekly dataset includes 4 training sets and 8 testing sets.

In the process of fingerprint data collection, the experimenter placed a laptop on a cart about 1 m above the ground and measure RSSI according to the collection direction. To avoid errors caused by chance, the experimenter continuously measures 10 sets of fingerprints at each RP or TP. The dataset of the underground parking lot contains a total of 20,400 ((45+41+21×4)×10×2×6=20400) wi-fi fingerprints, and each RP has a total of 120 (10×2×6=120) wi-fi fingerprints. The dataset of the underground parking lot is stored in the form of files, and the weekly data are stored in different folders. There are 4 files in CSV format for each test set or training set, holding fingerprint, location, time, and unique identifier data, respectively. Next, we use the underground parking lot fingerprint dataset for follow-up experiments.

### 4.2. Data Pre-Processing

In actual positioning, the RSSI presents strong fluctuation. In order to establish a robust fingerprint database, the general method is to collect RSSI multiple times at each RP within a certain period of time and remove abnormal RSSI. This paper uses 3σ criterion to find abnormal RSSI and replaces the abnormal value with the mean of RSSI sequence.

The vector rss is all the RSSIs from $AP_{i}$ measured at $RP_{j}$:

(28) rss=[R 1SSIAPiRPj,R 2SSIAPiRPj,…,R pSSIAPiRPj].

Calculate the residual error γ for each element of rss and standard error σ of rss:

(29) γk=R kSSIAPiRPj−1p∑ik=1pR ikSSIAPiRPj,k∈[1,p]

(30) $$\sigma =\sqrt{\left(\frac{1}{p-1}\sum_{k=1}^{p}\gamma_{k}^{2}\right)},$$

where R kSSIAPiRPj represents the RSSI for the k-th time from $AP_{i}$ measure at $RP_{j}$, $\gamma_{k}$ represents the residual error of R kSSIAPiRPj, and σ is the standard error of rss.

If $\left|\gamma_{k}\right|>3\sigma$, the R kSSIAPiRPj is judged to be a gross error value and should be replaced with the mean value of rss:

(31) R kSSIAPiRPj=1p∑ik=1pR ikSSIAPiRPj.

### 4.3. EMDT Experiment

#### 4.3.1. EMDT Smoothing RSSI Sequence

Before implementing the EMDT, it is necessary to construct the RSSI sequence X(t). In the offline sampling process, the RSSI sequence Xijoff(t) consists of the preprocessed RSSIs from $\;AP_{i}\;$ measured at $\;RP_{j}$, and each Xijoff(t) has a total of 120 RSSI data. The Xijoff(t) is shown in Equation (32):

(32) Xijoff(t)=R tSSIAPiRPj,t∈[1,120].

In the online positioning process, the RSSI sequence Xijon(t) consists of the RSSI from $AP_{i}$ continuously measured at $TP_{j}$, and each Xijon(t) has a total of 10 RSSI data. The Xijon(t) is shown in Equation (33):

(33) Xijon(t)=R tSSIAPiTPj,t∈[1,10].

**Figure 8:**
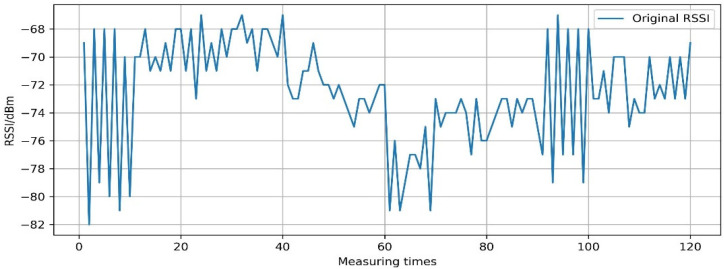
The original RSSI value of X10off(t).

Taking the RSSI sequence X10off(t)=R tSSIAP1RP0,t∈[1,120] as an example, the process of smoothing RSSI fluctuation by EMDT is explained in the following sentences. Figure 8 is the visualization of the original RSSI value of X10off(t). It can be seen from Figure 8 that the RSSI measured at the same RPs always fluctuates. The RSSI fluctuates sharply at measuring times [0,10] and [90,100], and the fluctuation range is about 12 dBm. The RSSI fluctuation frequency is relatively slow at the measuring times [10,90] and [100,120].

**Figure 9:**
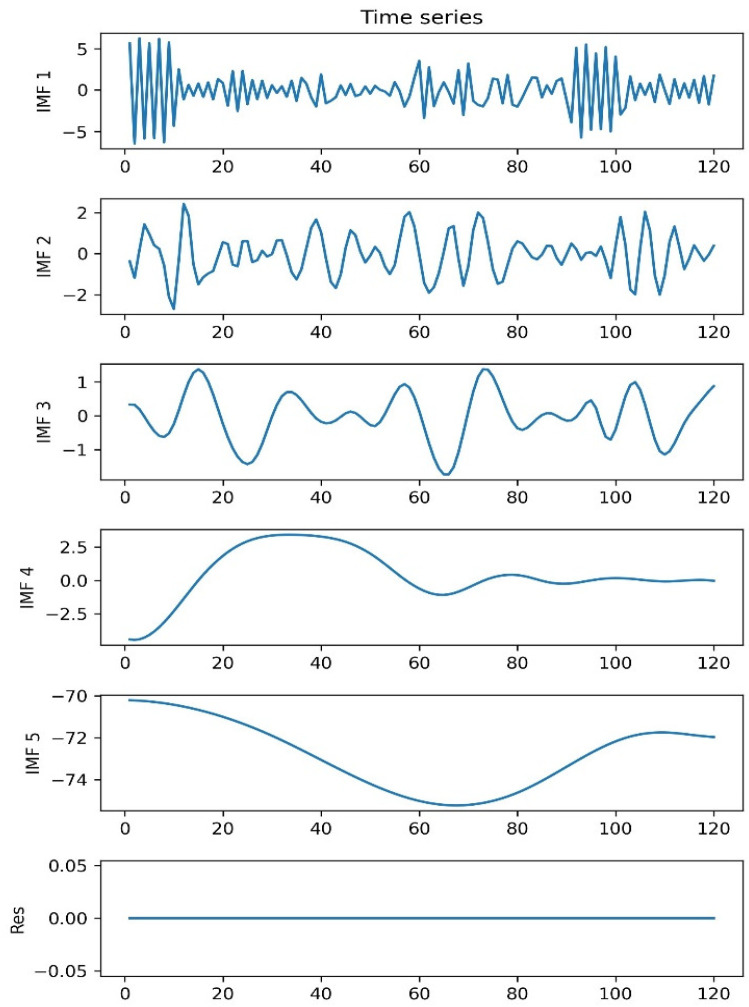
5 IMFs and a residual function.

After EMD decomposition, X10off(t) is decomposed into 5 IMFs and a residual function, and the decomposition result is shown in Figure 9. The frequency of each IMF decreases as the order number of the IMF increases, and $imf_{5}$ is the basically conforms to the fluctuation trend of X10off(t).

According to the IMF selection criteria proposed in this paper, IMFs are divided into FD-IMFs and E-IMFs. Table 2 lists the fluctuation energy $E_{\;}$, the fluctuation coefficient $K_{\;}$, and the possible fluctuation-only energy ${\hat{E}}_{\;}$ of all IMFs.

**Figure 10:**
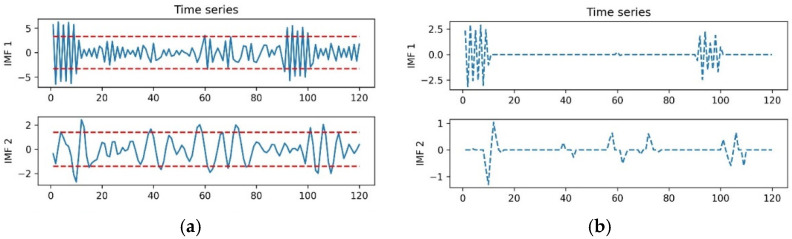
Soft threshold results. (**a**) The soft threshold in $imf_{1}$ and $imf_{2}$. (**b**) Threshold-based smoothed results in $imf_{1}$ and $imf_{2}$.

It can be seen that $E_{1}>{\hat{E}}_{1},E_{2}>{\hat{E}}_{2}$ and $\;E_{3}<{\hat{E}}_{3}$. According to the aforementioned, $imf_{1},imf_{2}$ are FD-IMFs and $imf_{3},imf_{4},imf_{5}$ are E-IMFs. Then the thresholds $TH_{1},TH_{2}$ are calculated to smoothing the $imf_{1},imf_{2}$. In Figure 10a, the blue curves are the decomposition results of $imf_{1}$ and $imf_{2}$, and the red lines are the soft thresholds of $TH_{1}$ and $TH_{2}$. The soft threshold-based smoothed results in $imf_{1}$, $imf_{2}$ are depicted in Figure 10b.

**Figure 11:**
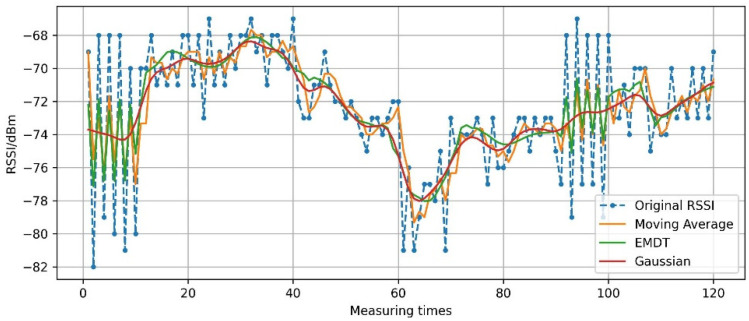
RSSI value smoothed by EMDT.

The RSSI sequence is reconstructed by smoothed $imf_{1},imf_{2}$ and $imf_{3},imf_{4},imf_{5},\;res$, and the smoothed RSSI sequence is show in Figure 11. In addition, Gaussian filter and moving average filter are performed on X10off(t), and the result is shown in Figure 11. All three methods can smooth RSSI fluctuation. At measuring times [0,10] and [90,100], the RSSI fluctuates sharply, and the smoothing effects of EMDT and moving average filter are basically the same; the Gaussian filter is too smooth to keep the difference of RSSI. At measuring times [10,90] and [100,120], the fluctuation frequency of RSSI is relatively slow, and the smoothing effects of EMDT and Gaussian filter are basically the same; the moving average filter is less smooth. It can be seen that the EMDT algorithm is able to smooth the data according to the degree of RSSI fluctuation and preserve the difference of RSSI.

#### 4.3.2. Processing of Outliers −105 dBm

**Figure 12:**
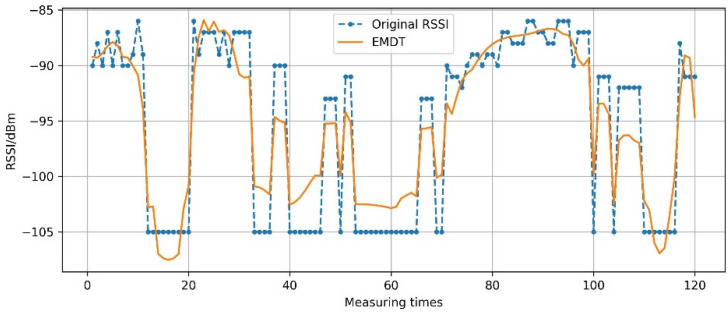
Abnormal situation.

The RSSI value will gradually attenuate with the increase of the propagation distance during the sign propagation process. Because of the equipment’s limitations, generally when the RSSI value is lower than −95 dBm, it is difficult to be measured by the equipment, that is, if RSSI<−95 dBm, it means that the network signal coverage is very poor, and there is almost no signal [36]. In this experiment, the unmeasured signal strength is taken as −105 dBm. The −105 dBm represents the RSSI, which is useless for improving the positioning accuracy. However, if the RSSI sequence X(t) containing −105 dBm is just processed by EMDT method, the smoothed RSSI sequence may have abnormal values. As shown in Figure 12, EMDT is performed on the RSSI sequence $X{\left(t\right)}_{off}=RSSI_{AP_{5}}^{RP_{0}}\left(t\right),t\in \left[1,120\right]$, and the smoothed RSSI values exceed −105 dBm.

In addition, it is inappropriate to perform EMDT smoothing on the RSSI sequence containing −105 dBm, because −105 dBm does not represent the RSSI real value. In this paper, the −105 dBm is replaced with the previous measuring RSSI value before smoothing the RSSI sequence containing −105 dBm.

### 4.4. Positioning Results and Comparison

#### 4.4.1. Impact of EMDT

**Figure 13:**
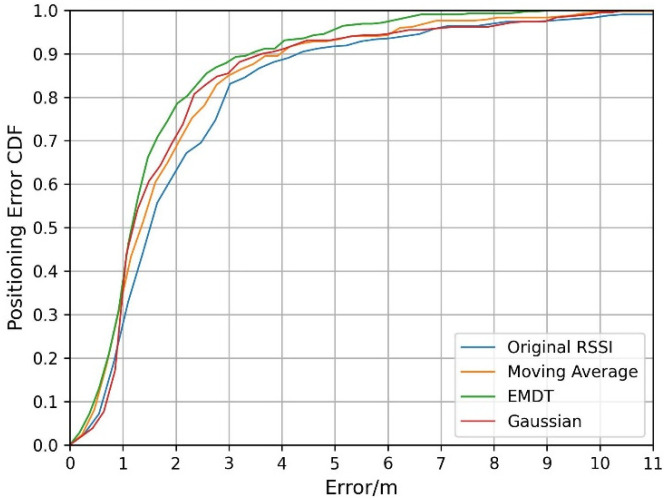
The positioning errors of CDF for different smooth methods.

Different smoothing fluctuation methods improve the positioning accuracy differently. We conducted comparative localization experiments on the original RSSI, Gaussian filter, moving average filter, and EMDT methods. The testing data is the first testing dataset of the first week. The cumulative distribution function (CDF) of positioning errors is shown in Figure 13. In this experimental dataset, the highest positioning accuracy based on the original RSSI and WKNN method is achieved when K is taken as 10, which is because TP and RP in this experiment do not overlap, and the minimum distance between them is 1 m.

It can find that all three methods of smoothing RSSI fluctuation can improve the positioning effect, and the EMDT positioning effect is the best. Within the positioning accuracy of 1 m, the Gaussian positioning effect is poor because when the RSSI fluctuates sharply, the Gaussian filter cannot maintain the difference between the RSSIs, resulting in the matched RPs away from the TP. Within the positioning accuracy of 2~6 m, the positioning effects of Gaussian and moving average are better than the original RSSI, indicating that smoothing the RSSI fluctuation can improve the positioning accuracy. The EMDT localization effect in Figure 13 is better than Gaussian and moving average, illustrating the importance of maintaining RSSI differences on the basis of smooth fluctuation.

#### 4.4.2. Impact of the Improved WKNN

The improved WKNN positioning algorithm uses the secondary selection method to remove the matching RPs far from the geometric center of the K initial matching RPs and constructs the combined weights to predict the TP coordinates. The improved WKNN positioning algorithm makes the predicted TP coordinates closer to the true TP coordinates. Therefore, it is very important to determine the distance threshold D for the secondary selection.

**Figure 14:**
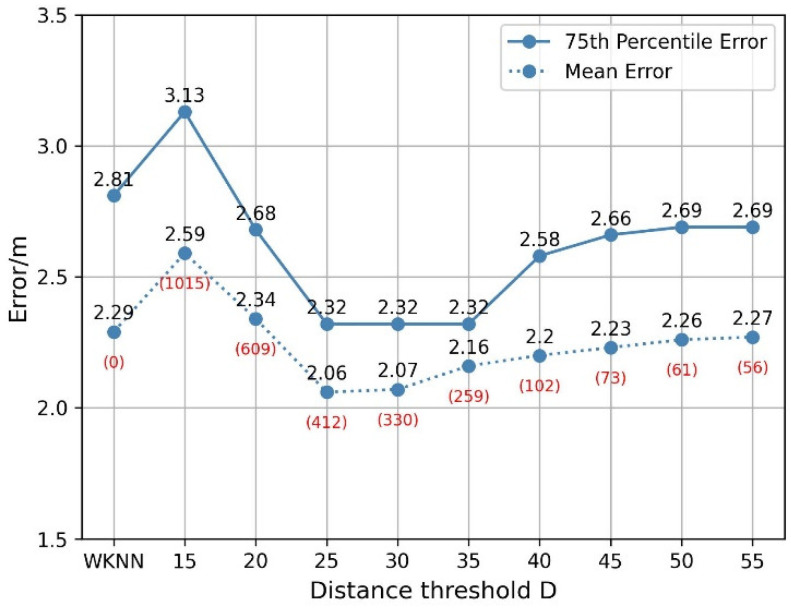
Positioning error for different thresholds.

The testing data is the first testing dataset of the first week. There are 210 test fingerprints in this experiment. Before the secondary selection, each TP will obtain 10 initial matching RPs by WKNN method, so a total of 2100 initial RPs will be matched. Figure 14 shows the 75th percentile positioning error and mean positioning error for different distance threshold values, and the D ranges from 15 to 55. The positioning error is the Euclidean distance between the actual coordinates and the predicted coordinates, and the mean positioning error is the mean Euclidean distance. The 75th percentile positioning error and mean positioning error of the WKNN are 2.81 and 2.29, respectively. When D≤15, about half of the matching RPs are removed, and the 75th percentile positioning error and mean positioning error rise compared to the WKNN. As the D value increases, the 75th percentile positioning error and mean positioning error continue to fall. When the D is 25, about 20% of matching RPs are judged as deviated RPs, and the 75th percentile positioning error and the mean error are minimized, which are 2.32 and 2.06 respectively. When the D is 25~35, the number of deviated RPs removed decreases, and the mean positioning error gradually increases again. Until D≥40, less than 5% of matching RPs are judged as deviated RPs, the 75th percentile positioning error gradually increases, but is still smaller than the WKNN algorithm. This result indicates that the matching RPs exist deviated RPs far away from the TP. In this paper, we set the distance threshold D to 25. The value of the D is closely related to the fingerprint database, and the most suitable threshold value needs to be calculated according to the actual situation.

#### 4.4.3. Impact of EMDT-WKNN

**Figure 15:**
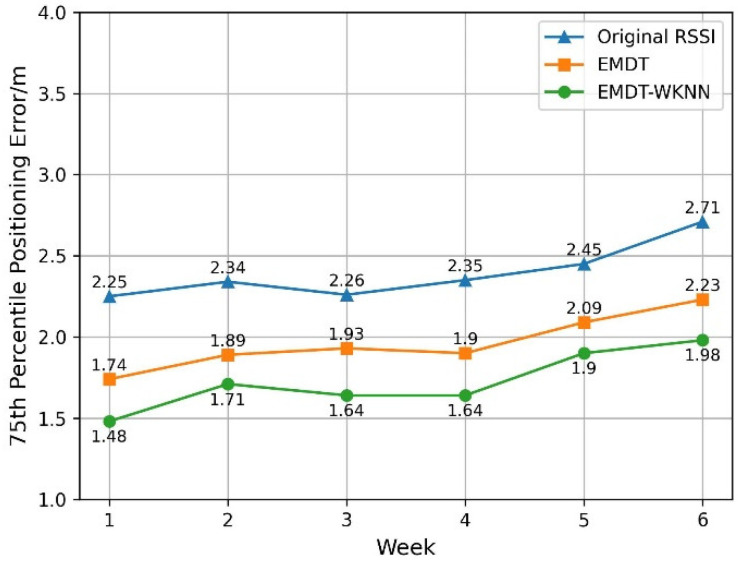
The 75th percentile positioning error for different weeks.

In the experiment, the wi-fi fingerprint positioning based on the Original RSSI, EMDT, and EMDT-WKNN was implemented respectively on the underground garage dataset of North China Electric Power University. The weekly 75th percentile positioning error is shown in Figure 15.

It can be seen from Figure 15 that, compared with the original RSSI, the 75th percentile positioning error of EMDT-WKNN achieves a decrease of 0.77 m, 0.63 m, 0.62 m, 0.71 m, 0.55 m, and 0.72 m, respectively. The average six-week 75th percentile localization errors of EMDT-WKNN and original RSSI are 1.73 m and 2.39 m, respectively, and the localization error of EMDT-WKNN achieved a 0.66 m drop, which is a 27.6% decrease. On the six-week testing dataset, the weekly 75th percentile positioning error of EMDT-WKNN is the smallest, and the improvement range is 0.55 m to 0.77 m, which shows the stability of the EMDT-WKNN method.

**Figure 16:**
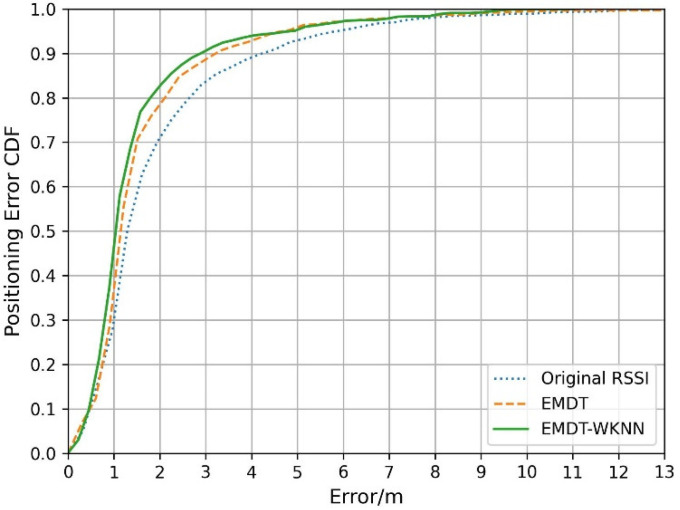
CDF of positioning error.

According to the experimental data of the first week, the CDF of positioning errors is shown in Figure 16. It can be seen that the positioning accuracy of EMDT-WKNN is better than EMDT and the original RSSI. Table 3 and Table 4 respectively compare the positioning accuracy of these three methods from different aspects.

Table 3 shows the cumulative error probability of the original RSSI, EMDT and EMDT-WKNN under a fixed precision limit. The 1 m, 2 m, and 3 m respectively represent the cumulative probability that the positioning error is less than 1 m, 2 m, and 3 m. Compared with the original RSSI, the positioning effect of EMDT is improved by 2.24%, 12.31%, 7.92%, 6.84%, and 5.42% in cumulative probability. It can be seen that the positioning effect of EMDT algorithm is significantly improved within 1 m to 3 m. Compared with the EMDT, the positioning effect of EMDT-WKNN is improved by 10.48%, 4.88%, 4.22%, 2.15%, and 2.26% in cumulative probability. The EMDT-WKNN positioning effect is significantly improved within 1 m. It shows that when most of the matching RPs are close to the actual position of TP, but some RPs are far away from the TP, EMDT-WKNN can effectively remove these deviation RPs, and the positioning effect is greatly improved.

Table 4 shows the positioning errors under different measurement metrics. The 68th and 95th percentile positioning errors correspond to the basic positioning error and the worst positioning errors [37]. Compared to the original RSSI, the mean error, the 68th percentile positioning error, the 75th percentile positioning error, the 95th percentile positioning error, and the standard deviation (SD) based on the EMDT-WKNN decreased by 21.2%, 27.2%, 34.2%, 22.3%, and 25.9%, respectively. The results show that the mean error, the 75th percentile positioning error and the SD are reduced, indicating the stability and effectiveness of EMDT-WKNN in improving localization performance.

## 5. Conclusions

We proposed an improved Wi-Fi indoor positioning method named EMDT-WKNN for smoothing RSSI fluctuation. EMDT-WKNN consists of three steps to improve the positioning accuracy. First, the EMDT is introduced to smooth the RSS fluctuation. Secondly, the secondary selection method is adopted to remove the RPs far from the geometric center of the K initial matching RPs. Finally, weights are calculated by combining the Euclidean distance of the matching RPs and the fingerprint similarity metric. In the positioning experiment of the underground garage of North China Electric Power University, the mean of the six-week 75% probability positioning error based on EMDT-WKNN is 1.73 m, and the positioning accuracy is improved by 27.6% compared with the original RSSI; the probability of positioning error based on EMDT-WKNN method within 3 m reached 90.71%. The results show that the indoor positioning method based on EMDT-WKNN simply and effectively smoothed the RSSI fluctuation and improved the positioning accuracy.

However, the experimental data in this study only comes from the underground garage dataset, and subsequent experiments will be carried out on other public datasets or different scenes to further validate its improvement.

## Figures and Tables

**Table 1:** Weekly data collection time.

Time	Monday	Tuesday	Wednesday	Thursday	Friday
14:00	Test_1	Test_3	Test_5	Train_1	Train_3
19:00	Test_2	Test_4	Test_6	Train_2	Train_4

**Table 2:** The E, K, and ${\hat{E}}_{\;}$ of each IMF.

IMF	$E_{i}$	$K_{i}$	${\hat{E}}_{i}$
$imf_{1}$	2.06	0.89	1.84
$imf_{2}$	0.85	0.39	0.80
$imf_{3}$	0.53	0.27	0.56
$imf_{4}$	1.22	0.73	1.51
$imf_{5}$	1.98	0.62	1.28

**Table 3:** Cumulative error probability of different algorithms under fixed accuracy limit.

Algorithm	1 m	1.5 m	2 m	2.5 m	3 m
Original RSSI	28.05%	58.04%	70.53%	78.45%	83.03%
EMDT	30.29%	70.35%	78.45%	85.29%	88.45%
EMDT-WKNN	40.77%	75.23%	82.67%	87.44%	90.71%

**Table 4:** Positioning errors under different measures metrics.

Algorithm	Mean Error (m)	68% Error (m)	75% Error (m)	95% Error (m)	Error SD (m)
Original RSSI	1.93	1.84	2.25	5.82	1.89
EMDT	1.62	1.41	1.74	4.61	1.61
EMDT-WKNN	1.52	1.34	1.48	4.52	1.48

## Data Availability

The experiment uses an internal data set. The data presented in this study are available on request from the corresponding author.

## References

[1] Basiri A., Lohan E.S., Moore T., Winstanley A., Peltola P., Hill C., Amirian P., Figueiredo e Silva P. (2017). Indoor location based services challenges, requirements and usability of current solutions. Comput. Sci. Rev..

[2] Hatem E., Fortes S., Colin E., Abou-Chakra S., Laheurte J.M., El-Hassan B. (2021). Accurate and Low-Complexity Auto-Fingerprinting for Enhanced Reliability of Indoor Localization Systems. Sensors.

[3] García-Paterna P.J., Martínez-Sala A.S., Sánchez-Aarnoutse J.C. (2021). Empirical Study of a Room-Level Localization System Based on Bluetooth Low Energy Beacons. Sensors.

[4] Cahyadi W.A., Chung Y.H., Adiono T. Infrared indoor positioning using invisible beacon. Proceedings of the 2019 Eleventh International Conference on Ubiquitous and Future Networks (ICUFN).

[5] Shen M., Wang Y., Jiang Y., Ji H., Wang B., Huang Z. (2020). A New Positioning Method Based on Multiple Ultrasonic Sensors for Autonomous Mobile Robot. Sensors.

[6] Tong H., Xin N., Su X., Chen T., Wu J. (2020). A Robust PDR/UWB Integrated Indoor Localization Approach for Pedestrians in Harsh Environments. Sensors.

[7] Feng X., Nguyen K.A., Luo Z. (2022). A survey of deep learning approaches for WiFi-based indoor positioning. J. Inf. Telecommun..

[8] Kitt B., Geiger A., Lategahn H. Visual odometry based on stereo image sequences with RANSAC-based outlier rejection scheme. Proceedings of the 2010 IEEE Intelligent Vehicles Symposium.

[9] Jeong J.P., Yeon S., Kim T., Lee H., Kim S.M., Kim S. (2018). SALA: Smartphone-assisted localization algorithm for positioning indoor IoT devices. Wirel. Netw..

[10] Billa A., Shayea I., Alhammadi A., Abdullah Q., Roslee M. An overview of indoor localization technologies: Toward IoT navigation services. Proceedings of the 2020 IEEE 5th International Symposium on Telecommunication Technologies (ISTT).

[11] Pan H., Xiang Y., Xiong J., Zhao Y., Huang Z., Xiao X. (2021). Application of a WiFi/Geomagnetic Combined Positioning Method in a Single Access Point Environment. Wirel. Commun. Mob. Comput..

[12] Sinha R.S., Hwang S.-H. (2020). Improved RSSI-Based Data Augmentation Technique for Fingerprint Indoor Localisation. Electronics.

[13] Bullmann M., Fetzer T., Ebner F., Ebner M., Deinzer F., Grzegorzek M. (2020). Comparison of 2.4 GHz WiFi FTM- and RSSI-based indoor positioning methods in realistic scenarios. Sensors.

[14] Mahapatra R.K., Shet N.S.V. (2018). Localization Based on RSSI Exploiting Gaussian and Averaging Filter in Wireless Sensor Network. Arab. J. Sci. Eng..

[15] Chen Y., Zhou R., Teng J., Zhou H., Luan Q. (2019). Indoor positioning method based on adaptive correction of Manhattan distance. Navig. Position. Timing.

[16] Aiboud Y., Elhassani I., Griguer H., Drissi M. Rssi optimization method for indoor positioning systems. Proceedings of the 2015 27th International Conference on Microelectronics (ICM).

[17] Zafari F., Papapanagiotou I., Hacker T.J. A novel Bayesian filtering based algorithm for RSSI-based indoor localization. Proceedings of the 2018 IEEE International Conference on Communications (ICC).

[18] Zhang L., Tan T., Gong Y., Yang W. (2019). Fingerprint Database Reconstruction Based on Robust PCA for Indoor Localization. Sensors.

[19] Lin K., Chen M., Deng J., Hassan M.M., Fortino G. (2016). Enhanced fingerprinting and trajectory prediction for IoT localization in smart buildings. IEEE Trans. Autom. Sci. Eng..

[20] Nikoukar A., Abboud M., Samadi B., Güneş M., Dezfouli B. Empirical analysis and modeling of Bluetooth low-energy (BLE) advertisement channels. Proceedings of the 2018 17th Annual Mediterranean Ad Hoc Networking Workshop (Med-Hoc-Net).

[21] Lu W., Cheng Y., Fang S. A study of singular value decomposition for wireless LAN location fingerprinting. Proceedings of the 2016 IEEE Second International Conference on Multimedia Big Data (BigMM).

[22] Liu J., Jia B., Guo L., Huang B., Wang L., Baker T. (2022). CTSLoc: An indoor localization method based on CNN by using time-series RSSI. Clust. Comput..

[23] Zhou R., Yang Y., Chen P. (2021). An RSS transform—Based WKNN for indoor positioning. Sensors.

[24] Huang N.E., Shen Z., Long S.R., Wu M.C., Shih H.H., Zheng Q., Liu H.H. (1998). The empirical mode decomposition and the Hilbert spectrum for nonlinear and non-stationary time series analysis. Proc. R. Soc. London. Ser. A Math. Phys. Eng. Sci..

[25] Boudraa A.O., Cexus J.C. (2007). EMD-based signal filtering. IEEE Trans. Instrum. Meas..

[26] Lakshmi M.D., Murugan S.S., Padmapriya N., Somasekar M. Texture analysis on side scan sonar images using EMD, XCS-LBP and statistical co-occurrence. Proceedings of the 2019 International Symposium on Ocean Technology (SYMPOL).

[27] Kaemarungsi K., Krishnamurthy P. (2004). Modeling of indoor positioning systems based on location fingerprinting. IEEE Infocom 2004.

[28] Guo J., Zhen D., Li H., Shi Z., Gu F., Ball A.D. (2019). Fault feature extraction for rolling element bearing diagnosis based on a multi-stage noise reduction method. Measurement.

[29] Li G., Hu Y. (2019). An enhanced PCA-based chiller sensor fault detection method using ensemble empirical mode decomposition based denoising. Energy Build..

[30] Hu M., Zhang S., Dong W., Xu F., Liu H. (2021). Adaptive denoising algorithm using peak statistics-based thresholding and novel adaptive complementary ensemble empirical mode decomposition. Inf. Sci..

[31] Cheng Y., Wang Z., Chen B., Zhang W., Huang G. (2019). An improved complementary ensemble empirical mode decomposition with adaptive noise and its application to rolling element bearing fault diagnosis. ISA Trans..

[32] Mohguen W., Bekka R.E.H. (2017). Empirical mode decomposition based denoising by customized thresholding. Int. J. Electron. Commun. Eng..

[33] Lei S., Lu M., Lin J., Zhou X., Yang X. (2021). Remote sensing image denoising based on improved semi-soft threshold. Signal Image Video Processing.

[34] Xie X., Xu W., Huang C., Fan X. (2021). New islanding detection method with adaptively threshold for microgrid. Electr. Power Syst. Res..

[35] Yang H., Cheng Y., Li G. (2021). A denoising method for ship radiated noise based on Spearman variational mode decomposition, spatial-dependence recurrence sample entropy, improved wavelet threshold denoising, and Savitzky-Golay filter. Alex. Eng. J..

[36] Pomalo M., El Ioini N., Pahl C., Barzegar H.R. A data generator for cloud-edge vehicle communication in multi domain cellular networks. Proceedings of the 2020 7th International Conference on Internet of Things: Systems, Management and Security (IOTSMS).

[37] Nakamura M., Akiyama T., Sugimoto M., Hashizume H. 3d fdm-pam: Rapid and precise indoor 3d localization using acoustic signal for smartphone. Proceedings of the 2014 ACM International Joint Conference on Pervasive and Ubiquitous Computing: Adjunct Publication.

